# Evaluation of ‘In-Parlour Scoring’ (IPS) to Detect Lameness in Dairy Cows during Milking

**DOI:** 10.3390/ani14192870

**Published:** 2024-10-05

**Authors:** Jasmin Laschinger, Birgit Fuerst-Waltl, Lisa Fuerst, Sophie Linnenkohl, Robert Pesenhofer, Johann Kofler

**Affiliations:** 1 Clinical Department for Farm Animals and Food System Science, Clinical Center for Ruminant and Camelid Medicine, University of Veterinary Medicine Vienna, 1210 Vienna, Austria; jasmin.laschinger@vetmeduni.ac.at (J.L.); 01560554@students.vetmeduni.ac.at (S.L.); 2 Institute of Livestock Sciences, BOKU University, 1180 Vienna, Austria; birgit.fuerst-waltl@boku.ac.at; 3 Faculty of Informatics, TU Wien, 1040 Vienna, Austria; l.fuerst11@gmail.com; 4 Claw Trimming Practice Robert Pesenhofer, 8151 Hitzendorf, Austria; klaue234@gmail.com

**Keywords:** lameness, locomotion scoring, in-parlour scoring, dairy cows

## Abstract

**Simple Summary:**

This study aimed to investigate the potential of scoring dairy cattle standing in the milking parlour, known as ‘In-Parlour Scoring’ (IPS), as an alternative method to locomotion scoring. A total of 990 observations were conducted on 495 cows across eleven Austrian dairy farms equipped with herringbone, side-by-side, or tandem milking parlours by two investigators. The IPS indicators included shifting weight, claw conformation, and visible disorders of the distal limb. Locomotion scoring, using five different scores, was carried out on these 495 cows after the second round of IPS. The indicators of shifting weight, abnormal weight distribution, swollen heel, hock joint or interdigital space, skin lesions on the lateral hock, claw position score, digital dermatitis lesions, short dorsal claw wall, and hyperextension of one claw were determined to be useful in predicting lameness, defined as a locomotion score (LCS) ≥ 3. The ability to correctly designate a cow as non-lame (LCS ≤ 2) was calculated to be at least ≥ 96% (specificity). However, the ability to correctly predict a lame cow was only 24% or less (sensitivity). We conclude that a one-time IPS has limited suitability for lameness detection on Austrian dairy farms with herringbone, side-by-side, and tandem milking parlours.

**Abstract:**

The objective of this study was to evaluate the potential of ‘In-Parlour Scoring’ (IPS) as an alternative to locomotion scoring in herringbone, side-by-side, and tandem milking parlours in Austria. Between January and May 2023, a total of 990 observations were conducted on 495 cows across eleven Austrian dairy farms by two investigators working simultaneously but independently of each other. The observation criteria included shifting weight, claw conformation, and obvious disorders of the distal limb. Locomotion scoring was conducted on all cows within 24 h of assessment in the milking parlour using a scale of 1 to 5 (LCS 1: not lame; LCS 5: severely lame). Functional hoof trimming was performed within ten days after IPS. The following indicators were identified as useful for predicting lameness (LCS ≥ 3): shifting weight, abnormal weight distribution, swollen heel, hock joint or interdigital space, skin lesion on the lateral hock, claw position score, digital dermatitis lesions, short dorsal claw wall, and hyperextension of one claw. The reliability of the individual indicators for intra- and inter-rater assessment exhibited considerable variation ((weighted) kappa values: −0.0020–0.9651 and −0.0037–1.0, respectively). The specificity and sensitivity for the prediction of lame cows were calculated to be ≥ 96% and ≤ 24%, respectively. It was demonstrated that a one-time IPS has limited suitability for lameness assessment on Austrian dairy farms with herringbone, side-by-side, and tandem milking parlours.

## 1. Introduction

The early detection of lameness [1,2] and the administration of professional treatment are of paramount importance in order to avoid economic losses and to ensure the welfare of dairy cattle [3,4,5,6]. Consequently, the development of effective and user-friendly lameness detection methods is imperative.

Currently, the most common method for identifying lame cows is to observe their gait [7]. For instance, locomotion scoring according to Sprecher et al. [8], which assesses the cow’s back posture while standing and moving, is a widely used method. However, the practicality of locomotion scoring is limited due to inadequate infrastructure on some farms. This is exemplified by the lack of opportunities to observe standing cows in pasture-based systems, uneven and slippery floors in freestall barns, or the lack of space due to overstocking. Furthermore, time constraint in any housing system is a key limiting factor [1,9].

It is evident that regardless of the locomotion scoring system employed, assessment of lameness by experts identifies a markedly greater number of lame cows than is estimated [10,11,12] or even assessed by farmers [13]. A variety of factors have been identified as contributing to the underestimation of lameness. These include farmers’ unawareness of lameness, as well as a phenomenon known as “operational blindness” due to desensitisation to lame cows over an extended period [12,13,14] and an excessive workload that results in a lack of time. However, the most probable explanation for the underassessment is a lack of education and training in locomotion scoring [13].

Consequently, there is a necessity to establish a reliable, simple-to-use alternative to the current methods of lameness assessment that do not require additional time from the farmer. An alternative approach to gait assessment is the use of a stall lameness score (SLS) protocol, which involves the observation of tied, standing cows observed for indicators or behaviours associated with lameness. These include uneven weight-bearing, resting of feet, standing on the edge of a step, rotation of feet, or weight shifting [15]. A cow is defined as lame by the presence of two or more indicators. In comparing lameness based on SLS with a gait-based locomotion score (five-point scale, according to Winckler and Willen [16]), it was found that SLS underestimated the proportion of lame cows compared to locomotion scoring.

A further approach involved the observation of cows locked in stanchions for lameness indicators, including an arched back, widely placed hind limbs, cow-hocked stance, or a preference for one leg while standing [17,18]. A comparison of these parameters with a gait-based locomotion score [8] revealed that this method lacked sufficient sensitivity or specificity to be used as an alternative to locomotion scoring.

A recently published study by Werema et al. [9] investigated the efficacy of in-parlour scoring (IPS) in pasture-based husbandry systems in New Zealand. The researchers observed cows for the presence of lameness indicators (shifting weight, abnormal weight distribution, swollen heel or hock joint, and overgrown hoof) during milking in a rotary milking parlour and compared this to locomotion scoring (0–3 scale, according to the Dairy NZ system). The researchers proposed that IPS could potentially represent a viable alternative to locomotion scoring in pasture-based dairy cattle [9]. Nevertheless, further studies are required, including farms under Central European conditions with smaller herd sizes that often use parallel or herringbone milking parlours.

The objective of this study was to assess the practicability and reliability of IPS during milking in parallel, herringbone, or tandem milking parlours and to compare these findings with the data of subsequent locomotion scoring conducted on farms with freestall housing in Austria.

## 2. Materials and Methods

### 2.1. Farm Location and Animal Data

This study was conducted on eleven dairy farms, comprising a total of 632 cows, located in the provinces of Lower Austria and Styria. The farmers were either clients of the University Clinic for Ruminants or expressed an interest in participating in this study. The farms were managed by the farmers themselves, with no employees.

The mean herd size was 57 (range: 27–123) dairy cows per farm with a mean annual milk yield of 10.513 kg. The cows were housed in freestall barns with cubicles, with no access to pasture. All herds were all-year-round calving herds. The farms milked twice daily in a parallel, herringbone, or tandem milking parlour. During the farm visit, herd size, mean annual milk yield, mean age of the herd, and frequency of hoof trimming were recorded (Table 1).

Digital dermatitis was endemic in all herds, except for farm 6. The farm visits for this study were conducted between February and May 2023.

### 2.2. In-Parlour Scoring (IPS)

Each cow was scored twice in the milking parlour, once in the evening and once during the subsequent morning milking, by two observers, independently but simultaneously. The first observer was an experienced veterinarian (J.L.), while the second (S.L.) was a final-year student of veterinary medicine with limited practical experience.

Three weeks before the start of data collection on site, visits were made to two farms, where the student had been trained in IPS by the expert. The cow’s hind limbs were visually screened for lameness indicators, which are summarised in Table 2.

All indicators evaluated by Werema et al. [9] were included (shifting weight, abnormal weight distribution, swollen heel or hock joint, overgrown hoof, observed claw injury, swelling/separation around the coronary band), except for “arched back”.

The evaluation of the backline was not possible in the milking parlours due to visual constraints and was thus excluded from consideration prior to the commencement of data collection.

### 2.3. Locomotion Scoring

Locomotion scoring was conducted by a veterinarian with extensive experience in locomotion scoring and in bovine orthopaedics (J.L.). Locomotion was scored in accordance with the scoring system described by Sprecher et al. [8] based on the co-assessment of gait and the backline over the caudal thoracic and lumbar vertebrae, while the cow was standing and walking on a five-point scale from locomotion score (LCS) 1 to 5 (Table 3). Locomotion scoring was conducted on a single occasion subsequent to IPS. The cows were restrained in stanchions after milking for the assessment of the backline while standing. Each cow was released individually, and gait and back-line assessments were conducted while the cow was walking in the loose housing system.

### 2.4. Hoof Trimming

Functional hoof trimming was conducted within one week following the IPS and locomotion scoring by two experienced professional hoof trimmers. For this procedure, the cows were positioned on a tilting table. All the observed claw lesions were documented using an electronic documentation system (‘Klauenmanager’, SEG Informationstechnik GmbH, Bad Ischl, Austria).

Cow-level prevalences were calculated for ‘alarm lesions’, the acute stage (M2) of digital dermatitis, and all stages of digital dermatitis (skin lesion stages M1 to M4.1), white line disease, and foot rot by dividing the number of affected cows by the total number of cows examined.

In accordance with the classification proposed by Kofler et al. [19], claw lesions always associated with pain were designated as ‘alarm lesions’. The term ‘alarm lesions’ encompasses all ulcers (sole, toe, bulb ulcers), toe necrosis, white line abscess, inflammatory swelling of the coronet and bulbs of the heel associated with deep digital sepsis, penetrating infected horn fissure, interdigital phlegmon, acute (M2) stage of dermatitis digitalis (DD), and all DD-associated claw horn lesions. In cases where interdigital hyperplasia was observed in association with a DD infection, but no further precise classification was provided, the M2 stage was assumed.

### 2.5. Statistical Data Analysis

Sample size was calculated according to Buderer et al. [20], using the pre-determined values of sensitivity (0.9), specificity (0.9), and prevalence (30%) as well as the precision of the estimate (i.e., the maximum marginal error, 5%). Based on these assumptions, the sample size for sensitivity was 461 and for specificity it was 198. Thus, at least 461 cows were required.

Initial processing of the data was conducted using Microsoft Excel 2020 (Microsoft Corp., Redmond, WA, USA). Only data from cows with two IPS and one locomotion score, which were subsequently subjected to claw trimming, were included in the following analysis (495 out of 632). The frequency of each potential indicator was calculated to evaluate its overall relevance. In order to assess the reproducibility of the IPS, the intra-rater reliability of the IPS indicators between the evening and morning milking was calculated for the experienced observer. Furthermore, the inter-rater reliability of the IPS indicators between the experienced veterinarian and the final-year veterinary student was also calculated. The (weighted) kappa values, determined by using the procedure freq of SAS 9.4 (SAS Institute Inc., Cary, NC, USA), were interpreted in accordance with Landis and Koch [21], with values <0 indicating poor, 0–0.20 indicating slight, 0.21–0.40 indicating fair, 0.41–0.60 indicating moderate, 0.61–0.80 indicating substantial, and 0.81–1.00 indicating an almost perfect strength of agreement.

The capacity to predict the presence of moderate to severe lameness (locomotion scores ≥ 3 according to Sprecher et al. [8]) based on the IPS indicators was determined through the application of a decision tree machine learning method (DT) [22] implemented in Scikit-learn, which is based on Python 3.9 [23]. Following the analyses of Werema et al. [9], a four-fold cross-validation was performed. This means that the dataset was divided into four equally sized subsets (or folds). The model was trained on each combination of three subsets, and the remaining fourth sub-set was used for validation. Pruning, which reduces the size of decision trees by removing parts that do not provide significant predictive power [24], was based on the criteria that a minimum of 20 observations were required to split an internal node and that a split at a node had to decrease Gini impurity by at least 0.0025. The Gini impurity quantifies the probability of misclassification of a randomly selected element in the dataset, i.e., lower values are preferable.

For each of the four results, sensitivity (or recall), specificity, precision, accuracy, and the F1 score (harmonic mean of precision and sensitivity) were calculated [25]. An overview of all metrics used to evaluate the decision tree models and their calculations are given in Table 4.

Confusion matrices were created to visualise the correctly and incorrectly classified observations, i.e., true positives, false negatives, false positives, and true negatives. Further, graphical decision trees were also used to visualise results. Both visualisation methods are implemented in Scikit-learn.

All calculations were made for observations during the morning and evening milkings together and separately, as well as on a combined dataset comprising the maximum value of both.

## 3. Results

### 3.1. Reliability of In-Parlour Scoring

A total of 495 dairy cows were included in the following analysis. The mean duration of milking per farm was 79 min (min 60 min; max 125 min). The effective observation time per cow for the assessment of all 16 IPS indicators exhibited considerable variation, with a range of 30 to 150 s (average 97 s).

The results of the intra-rater reliability of the IPS indicators assessed at the evening and morning milkings are listed in Table 5. The IPS indicators DD around the dew claws, DD interdigital space, SDW, and HC demonstrated substantial-to-(almost)-perfect agreement, according to Landis and Koch [21]. The lowest level of agreement was observed for DD above the heel and DD on skin above the coronary band, with kappa values of less than 0.00. The remaining IPS indicators demonstrated fair-to-moderate agreement.

To ascertain the reliability of IPS when performed by an inexperienced individual, the inter-rater reliability of the IPS indicators between an experienced veterinarian and a final-year student of veterinary medicine was calculated (Table 6). The results demonstrated a (weighted) kappa value of 0.3 or above, except for DD above the heel and DD on skin above the coronary band. These findings can be interpreted as indicative of fair-to-(almost)-perfect agreement.

### 3.2. Distribution of Locomotion Scores and In-Parlour Scoring Indicators

The prevalence of lameness across all eleven farms was 59.7% for LCS ≥ 2 and 17.3% for LCS ≥ 3, respectively. It exhibited considerable variation on the eleven farms, with values ranging from 34.2% to 97.7% for LCS ≥ 2 and from 0% to 72.1% for LCS ≥ 3, respectively. The distribution of LCS, according to Sprecher et al. [8], for each farm is presented in Table 7. A total of 495 cows were included in the analysis, with each cow assigned three scores: one for locomotion and two for the observation in the parlour.

Due to the limited number of cows with a locomotion score of 4 or 5 (overall 4.2% and 0% of all scores, respectively), scores 4 and 5 were combined to create a single score (LCS ≥ 4).

A total of 16 IPS indicators (Table 2) were initially identified for consideration. Eight indicators (OH, OCI, SCB, CD, CC, IH, BB, and AP) were finally excluded from further consideration due to their lack of utility in the present study. All these indicators were observed with a low frequency (*n* = 2, 3, 1, 3, 5, 4, 7, 5, respectively) during the data collection process. Furthermore, CD and CC were deemed to be of limited value, as they do not typically result in lameness. Additionally, CD and IH could not be assessed on three and four farms, respectively, due to heavy soiling of the claws, poor lighting conditions in the parlour, or the positioning of the animals in the milking parlour.

The frequency and distribution of observations for the remaining indicators are presented in Table 8 (evening milking) and Table 9 (morning milking).

### 3.3. Association of In-Parlour Scoring Indicators and Locomotion Scores (Decision Tree Method)

**Figure 1:**
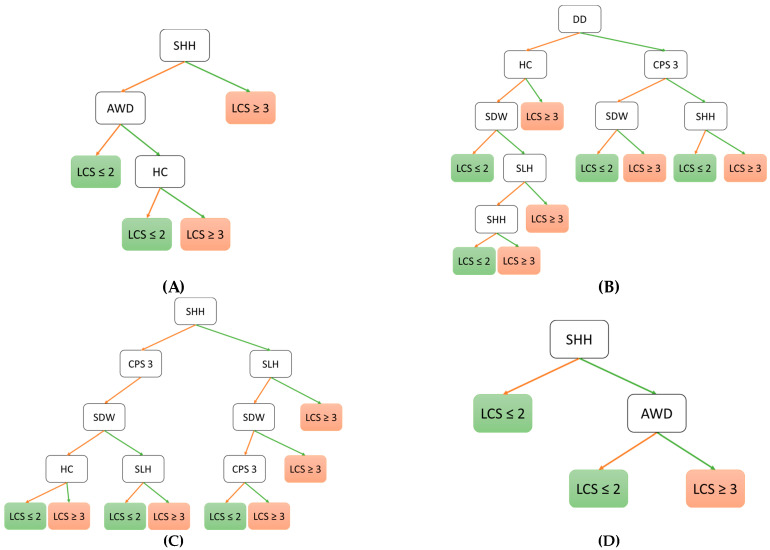
A decision tree (DT) to classify cows into locomotion score (LCS) ≤ 2 (non-lame to slightly lame) and LCS ≥ 3 (moderately-to-severely lame) using in-parlour scoring indicators of 990 observations across eleven farms. (**A**) DT based on observations of evening milking. (**B**) DT based on observations of morning milking. (**C**) DT based on observations of evening and morning milkings. (**D**) DT based on observations of evening and morning milkings combined to a maximum value. Orange arrow = absence of indicator; green arrow = presence of indicator; AWD = abnormal weight distribution; SHH = swollen heel, hock joint, or interdigital space; SLH = skin lesion on the lateral hock; CPS = claw position score; DD = digital dermatitis (acute M2 stage); SDW = short dorsal claw wall; HC = hyperextension of one claw.

**Figure 2:**
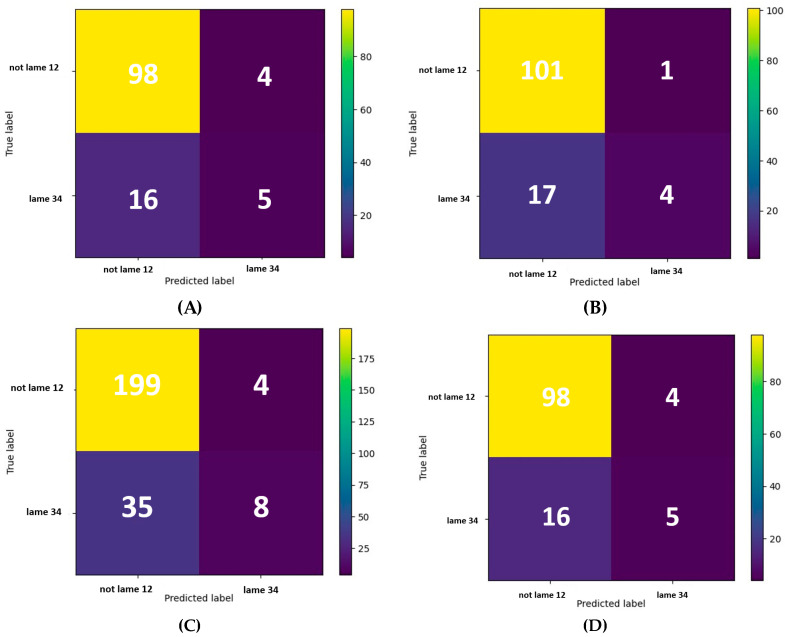
A Confusion matrix of the decision tree classifier with the highest test accuracy using eight in-parlour scoring indicators of 990 observations across eleven farms. (**A**) DT based on observations of evening milking. (**B**) DT based on observations of morning milking. (**C**) DT based on observations of evening and morning milkings. (**D**) DT based on observations of evening and morning milkings combined to a maximum value.

The classifiers with the highest test accuracy in each case are presented in Figure 1. F1 scores between 0.29 and 0.33 (Table 10) were calculated for these classifiers. Table 10 also provides a summary of sensitivity, specificity, precision, and accuracy for these classifiers. Figure 2 presents the confusion matrices, which illustrate the true and false positive rates, as well as the true and false negative rates.

### 3.4. Claw Lesions

Functional hoof trimming was performed within one week of the IPS. The prevalence of painful ‘alarm lesions’ associated with lameness [19] identified during the subsequent functional hoof trimming of the 495 cows, conducted by two professional claw trimmers, is presented in Table 11. A total of 173 ‘alarm lesions’ were identified in 133 out of 495 cows, representing a prevalence of 26.9% at cow level. The prevalence at cow level for the acute stage (M2) of digital dermatitis was 18.4%. The prevalences at cow level for white line disease, digital dermatitis (skin lesion stages M1 to M4.1), and foot rot were 39.0%, 26.1%, and 0.8%, respectively.

## 4. Discussion

Until automated lameness detection systems for dairy cows are not widely available for farmers and easy to use as currently commercially available sensors for automated heat detection [26,27,28], visual methods for early lameness detection remain indispensable [7,8,13]. However, visual identification of lame cows requires a certain amount of time for farmers who are in daily contact with their cows [8,12,14]. Therefore, we assessed the efficacy of the IPS developed by Werema et al. [9] in Austrian dairy farms utilising herringbone, side-by-side, or tandem parlours. Two observers (an experienced veterinarian and an inexperienced student) scored all cows simultaneously during two consecutive milkings on a total of eleven farms.

The lameness prevalence of 59.7% observed in this study was higher than reported in a recently published study in 144 Austrian dairy herds when cows with LCS 2 were classified as lame (50.2%), but lower than when animals were defined as lame with LCS ≥ 3 (17.3% vs. 31.2%) [29]. In contrast to Werema et al. [9], which classified 0.5% (10/4125) of the cows as severely lame, we assessed 4.2% (21 of 495) of the cows with the highest degree of lameness (LCS ≥ 4). This severe form of lameness, which is characterised by partial or complete absence of limb weight-bearing [8], is more readily identifiable in standing cows in the milking parlour than LCS 2 or LCS 3.

It should be noted that the evaluation was conducted on only eleven farms, and only those cows with data from two IPS, one locomotion scoring, and subsequent hoof trimming were subjected to further analysis. This could have led to a certain degree of selection bias.

### 4.1. Practicability of IPS

The primary challenges encountered during data collection were poor lighting in the parlour, time constraints during the IPS due to only 30 to 150 s per cow on average for the assessment of all 16 IPS indicators, and occasionally heavily soiled claws. Additionally, depending upon the milking parlour, certain parts of the claw or parts of the limb were poorly visible. For instance, in the tandem parlour, the hock joint could only be evaluated from the side facing the examiner. This may be a contributing factor to the relatively low intra-rater reliability of 0.5901 (0.4075–0.7727) for swollen hock joints and 0.5050 (0.4463–0.5638) for skin lesions on the lateral hock.

Other limiting factors were the lack of space for the cows in the milking parlour and the inclined standing area in some milking parlours (Table 1). This resulted in the animals adopting unphysiological limb positions, which were interpreted by the observers as relief positions and led to falsely high CPS.

The identification of the animals was not a significant issue in our study, as the farmer was able to readily identify the cows based on their appearance and udder characteristics due to the relatively small herd sizes. Furthermore, automatic animal identification was usually available in the milking parlours, and the cows wore collars bearing easily recognisable ID numbers. From our perspective, the time required to complete locomotion scoring and IPS is essentially equivalent. It is necessary to allocate sufficient time for both scoring systems. However, the advantage of IPS versus locomotion scoring is that the farmer must not spend additional time.

Arched back was excluded in advance due to the lack of visibility, which was also the case in the parallel, herringbone, or tandem milking parlours in our study, as also reported by Werema et al. [9] for the rotary milking parlour. Furthermore, this indicator would have been of limited value in the milking parlours, which were insufficiently spacious for the dimensions of the cows. A total of 16 IPS indicators (Table 3) were initially identified for consideration. Eight indicators were finally excluded from further analysis. This was either due to lack of usefulness, a low frequency, assessment only in some farms, or because some of them, e.g., CD and IH, do not typically lead to lameness [30,31]. Additionally, CD and IH could not be assessed on three and four farms, respectively, due to heavy soiling of the claws, poor lighting conditions in the parlour, or the positioning of the animals in the milking parlour. Despite its low frequency (*n* = 5), hyperextension of one claw (HC) was included in the further analysis, as this is frequently a characteristic sign of deep digital sepsis affecting the deep digital flexor tendon [32,33]. This is one of the ‘alarm lesions’ [19], as it is always associated with pain and thus lameness.

In contrast to the results of Werema et al. [9], which indicated that overgrown hooves (OH) were a useful indicator, our study did not identify OH as a predictor of lameness (LCS ≥ 3). However, short dorsal claw wall (SDW) was found to be a useful indicator, as shown by others [34]. The results of these studies [9,34] indicate that the length of the dorsal claw wall may be a useful indicator of lameness. However, it depends on the housing conditions whether overgrown hooves, as in pasture-based housing conditions, or short dorsal claw walls, as in loose housing systems, are more common [35]. As described by Werema et al. [9] and Schönberger et al. [36], our results confirmed that SW is a useful indicator for detecting lame cows in the milking parlour. One additional indicator considered was the acute stage of DD. Several studies have demonstrated that DD can be identified in the milking parlour [37,38,39,40]. Accuracy of detecting DD lesions in milking parlours can be improved by washing the claws beforehand [40], using a headlamp, and possibly a swivelling mirror [37,38]. In order to ascertain the suitability of IPS for practical use, we did not utilise any of these tools. Nevertheless, we found that acute DD was a suitable indicator for IPS. As reported by Werema et al. [9], the prevalence of swelling/separation around the coronary band (SCB) was low, with only one case identified due to the influence of external factors, such as dirt and poor lighting. The low incidence of OCI (observed claw injury) was also confirmed in our herds.

### 4.2. Reliability of IPS

The IPS indicators DD around the dew claws and in the interdigital space, SDW and HC exhibited substantial-to-(almost)-perfect agreement for the experienced observer with kappa-values of 0.8323, 0.6215, 0.9651, and 0.7990, respectively [21]. The remaining IPS indicators demonstrated fair-to-moderate levels of agreement with kappa-values of 0.2203 (SW), 0.2362 (AWD), 0.4985/0.5901/0.4422 (SHH), 0.5050 (SLH), and 0.3996 (CPS). The highest values of intra-rater reliability with weighted kappa values of 0.7990, 0.9651, and 0.8323 were calculated for HC, SDW, and DD around the dew claws. Possible reasons for the lower agreement of some indicators are that in herringbone or tandem parlours, most cows stand on different sides of the parlour at each consecutive milking, so lesions may be well seen at one milking and not on the next milking. This problem did not arise in the study of Yang and Laven [40], as the examined cows stood only on one side of the herringbone parlour, nor in the study of Werema et al. [9], as the cows were milked in a rotary milking parlour. Other studies also examined cows for DD on both sides of the herringbone milking parlour for lesions [37,38], but did not mention that the different sides of the milking parlour had an influence on the results. However, these studies only investigated DD lesions on the claws and not unilateral skin lesions on the lateral hock or similar as in our study. Of course, other factors may also play a role: cramped parlours, where cows are squeezed in differently [41]; depending on the cause of the relief position/tripping, e.g., subjectively, it seemed more pronounced in evening milkings when cows have already been on their feet all day. This could explain the higher sensitivity and the F1 score of the classifier of the evening milking compared to the morning milking (0.24, 0.33 vs. 0.19, and 0.31). The relatively short observation time per cow may also mean that some indicators were overlooked or not shown at the time of assessment [42]. In comparison to the study by Werema et al. [9], which had approximately 30 s to evaluate six IPS indicators on one cow, the two observers in our study had between 30 and 150 s on average to assess 16 indicators, with the exact time varying depending on the farm. The evaluation of IPS indicators during milking did not impede the farmers’ work processes or affect the duration of the milking process. The discrepancy in observation times per farm can be attributed solely to the efficiency of the respective farmers in milking.

Comparing the assessments of the experienced observer with those of the inexperienced observer, the results showed a (weighted) kappa value of 0.3 and above, except for DD above the heel and DD on skin above the coronary band. These findings can be interpreted as indicative of fair-to-almost perfect agreement [21]. There was a poor agreement between the two observers for DD above the heel (kappa −0.0037) and DD on the skin above the coronary band (kappa 0.0000). In other studies [37,43] higher levels of agreement between observers with kappa values of 0.51 and >0.74 were calculated, respectively, attributing DD scores. However, these researchers inspected cleaned feet with a swivelling mirror and a powerful headlamp in the milking parlour. It has been found that there is a 93.9% probability that the sensitivity of the examination for DD post-washing is greater than that pre-washing [40]. Washing the claws would therefore also have led to better results in our study, as many claws were heavily soiled and therefore difficult to assess. However, even among experienced European observers, there was only moderate agreement for the M scores (Gwet’s agreement coefficient = 0.48), indicating a degree of individual variation [44]. The best agreement with kappa values of 1.000 (HC) and 0.9463 (SDW) between the two observers could be achieved for hyperextension of one claw and short dorsal claw horn wall. Since the length of the dorsal claw horn is directly and linearly correlated with the sole thickness [34,45,46], the length of the dorsal wall must be adjusted by functional hoof trimming for the age and breed of the cow [47]. A dorsal wall length of less than 7.5 cm was defined as too short, and over 10 cm as overgrown [34,45]. These indicators appear to be easily recognisable and obvious even to untrained observers and without a measuring template. Furthermore, these parameters do not depend on the time of the assessment, as they are always constant. This is different, for example, in the case of the relief position or weight shifting, where it depends very much on the exact moment of the IPS whether an indicator can be perceived or not. Although the two observers attempted to score the individual cows in the milking parlour as simultaneously as possible, this was hardly possible due to the usually very cramped conditions in the milking parlour, where the farmer as a third person was also present.

### 4.3. Assessment of IPS as a Method of Detecting Lame Cows (LCS ≥ 3)

The calculated F1 scores between 0.29 and 0.33 for the best and even lower mean F1 scores for all four test splits (Supplementary Material) indicate poor reliability for the classifiers. Depending on the observations used, high specificities (≥96%) but low sensitivities (≤24%) to determine locomotion scores could be calculated for the classifiers. Depending on the initial data, 84–85% of the observations in the test data could be correctly classified. This is lower compared to the results of Werema et al. [9] with 96.6%. This could be due to the different types of milking parlours, but it is also conceivable that the locomotion scoring system used played a role. In contrast to Werema et al. [9], who used four-level (score 0–3) locomotion scoring according to the DairyNZ system [48], we used the modified four-level (LCS 1–4) [13] locomotion scoring system of Sprecher et al. [8]. In addition, we did not assess the gait of the cows when they left the milking parlour, but after the second IPS in the loose housing the next morning, whereby the animals were initially fixed in the feed fence to assess their backline. One of the principal differences between the two studies is the number of farms and types of milking parlours involved, as well as the frequency of in-parlour scoring and locomotion scoring. Our study encompassed eleven farms with three distinct milking parlours and limited space. In-parlour scoring of the same cows was conducted only twice, and locomotion was scored only once. In contrast, Werema et al. [9] focused on two similar farms that used the same milking parlour system (rotatory milking parlour) and maintained a distance of about 1 m from the cows. Additionally, they conducted IPS and locomotion scoring of the same cows over a period of nine months, which could have influenced their results. Another difference between our study and the study of Werema et al. [9] is the prevalence of digital dermatitis. In our eleven farms, the prevalence at cow level was 26.1%, while the two farms in the New Zealand study had no cases of DD.

Different locomotion scoring systems may have influenced results due to different evaluation criteria. The DairyNZ lameness score is based on a comprehensive assessment of multiple physical characteristics, including walking speed, walking rhythm, weight-bearing, back alignment, head position, stride length, and foot placement [48]. The locomotion scoring system of Sprecher et al. [8] is based on the assessment of the backline while the cow is standing and walking, as well as on the cow’s gait. However, the assessment of locomotion is inherently subjective, which results in a lower inter-rater agreement than intra-rater agreement [49]. Furthermore, the result of locomotion scoring could have been influenced by the physical contact when releasing the animals from the feed fences or when herding them along the corridor. Physical handling produces stress, which may reduce observed pain-related behaviours [50]. The slippery floors, steps, and edges in the loose housing or the avoidance of higher-ranking animals in some stables could also have falsified the result [51,52]. In contrast to Werema et al. [9], our analysis included animals that were already undergoing treatment for claw lesions. Furthermore, due to the small amount of data in our study, it was not possible to analyse the data for herringbone, side-by-side, and tandem parlours separately.

Further studies are needed to find a way to obtain high sensitivity and specificity at the same time. This might be achieved by applying IPS twice daily for two or three consecutive days, as the combination of data from evening and morning milkings achieved the highest sensitivity, specificity, precision, and accuracy for the classifiers. Another approach could be to include only a few, but larger Austrian dairy farms with the same type of milking parlour and an adequate size of the milking parlour length in relation to the size of the cows.

## 5. Conclusions

The present study has shown that a one-time IPS is only partially suitable to predict lameness (LCS ≥ 3) on Austrian dairy farms with herringbone, side-by-side, and tandem milking parlours (specificities ≥ 96% and sensitivities ≤ 24%). Indicators SW, AWD, SHH, SLH, CPS, DD, SDW, and HC were considered useful. However, the intra-rater reliability varied widely (−0.0020–0.9651). The reproducibility of the results between different observers also varied between −0.0037 and 1.0.

## Figures and Tables

**Table 1:** Characteristics of the eleven participating dairy farms.

Farm	Herd Size (Number of Cows Analyzed)	Mean Annual Milk Yield (kg)	Dominant Breed	Mean Age of Herd (Years)	Milking Parlour Type (Units)	Hoof Trimming Frequency
1	73 (38)	9729	FV	5.5	Tandem * (2 × 4)	2–3 times a year (F)
2	40 (35)	9427	HF	4.3	Side-by-Side ^#^ (1 × 6)	3 times a year (HT)
3	123 (109)	13,801	HF	4.1	Side-by-Side (2 × 20)	3 times a year (HT)
4	83 (71)	10,195	FV	6.3	Side-by-Side * (2 × 8)	2 times a year (F)
5	39 (34)	10,668	HF	5.1	Herringbone (2 × 4)	Every 9 months (HT)
6	65 (18)	9362	FV	5.0	Side-by-Side *^#^ (2 × 6)	2 times a year (HT)
7	27 (25)	10,539	FV	6.5	Herringbone (1 × 4)	2 times a year (HT)
8	35 (32)	10,732	FV	5.0	Herringbone (2 × 3)	2 times a year (HT)
9	52 (46)	10,683	FV	5.1	Herringbone (2 × 7)	2 times a year (HT)
10	48 (44)	10,058	FV	5.5	Herringbone (2 × 4)	3 times a year (HT)
11	47 (43)	10,453	BS	5.5	Herringbone (1 × 4) Tandem (1 × 2)	3 times a year (HT)

* = milking parlour was too small for the size of the cows/cows, which were squeezed in the milking parlour and could not stand normally. ^#^ = standing area in the milking parlour with up to 5% gradient; FV: Fleckvieh (dual purpose Simmental); HF: Holstein–Friesian; BS: Brown Swiss; F: farmer; HT: hoof trimmer.

**Table 2:** The list of indicators used during the in-parlour scoring procedure. The indicators were adopted by Werema et al. [9] and supplemented.

Indicators	Description
Shifting weight (SW)	Frequent changing of feet during evaluation
Abnormal weight distribution (AWD)	The asymmetric placing of the claws on the ground
Swollen heel, hock joint, or interdigital space (SHH)	Abnormal swelling of the heel and surrounding tissues (observed from the plantar aspect of the foot), hock joint (lateral aspect), or immediately above the interdigital space
Overgrown hoof (OH)	Length of dorsal hoof wall > 10 cm on at least one hind limb
Observed claw injury (OCI)	Observation of claw injury of any type, i.e., cuts
Swelling/separation around the coronary band (SCB)	Abnormal swelling or separation around the coronary band
Skin lesion on the lateral hock (SLH)	Reddening or hairless area on the lateral aspect of the hock joint
Claw position score (CPS)	Evaluates the external rotation of the interdigital axis in relation to the body centerline <17 degrees (score 1) 17–24 degrees (score 2) >24 degrees (score 3)
Concave dorsal wall (CD)	Observation of concave dorsal wall of the claw as a sign of chronic laminitis on at least one claw
Corkscrew claw (CC)	The mid and caudal areas of the abaxial wall curve ventrally and can become part of the bearing surface of the claw; Axial displacement of the sole and axial white line and rotation of the toe; The toe and axial bearing surface becomes non-weight bearing
Interdigital hyperplasia (IH)	Small and painless protrusion of the interdigital skin or firm tumour like masses in the interdigital space
Digital dermatitis lesion (DD)	Observation of digital dermatitis lesions (M1, M2, M3, M4, or M4.1) on at least one claw
Short dorsal claw wall (SDW)	Due to the direct linear correlation of the dorsal claw horn length and the sole thickness, a thin sole was diagnosed by a short dorsal wall (<7.5 cm).
Bandages or blocks (BB)	Bandages or blocks attached to at least one claw
Abscesses/swelling on proximal limb (AP)	Abnormal swelling of the soft tissue proximal to the tarsal joint
Hyperextension of one claw (HC)	Upward tilting of the tip of a claw, indicating that the deep digital flexor tendon is no longer intact

**Table 3:** Description of locomotion scoring according to Sprecher et al. [8].

LCS	Clinical Term	Valuation Criteria
1	Sound	Stands and walks normally with a level back. Makes long confident strides. All feet placed with purpose.
2	Mildly lame	Stands with flat back, but arches when walks. Gait is slightly abnormal.
3	Moderately lame	Stands and walks with an arched back and short strides with one or more legs.
4	Lame	Arched back standing and walking. One or more limbs favoured but at least partially weight bearing.
5	Severely lame	Arched back, refuses to bear weight on one limb. May refuse or have great difficulty moving from lying position.

**Table 4:** Metrics used for evaluating decision tree models.

Metrics	Calculation
Sensitivity	TP/(TP + FN)
Specificity	TN/(FP + TN)
Precision	TP/(TP + FP)
Accuracy	(TP + TN)/(P + N)
F1 score	2TP/(2TP + FP + FN)

TP = true positives; FN = false negatives; TN = true negatives; FP = false positives; P = all positives; N = all negatives.

**Table 5:** The intra-rater reliability of the in-parlour scoring (IPS) indicators for the experienced observer is presented with a 95% confidence interval (in brackets) to demonstrate the reproducibility of this examination procedure. This analysis encompassed observations across eleven farms, collected during morning and evening milkings.

IPS Indicators	Intra-Rater Reliability
SW (*n* = 495)	0.2203 (−0.0051–0.4458)
AWD (*n* = 990)	0.2362 (0.1721–0.3003)
SHH	
Swollen heel (*n* = 1980)	0.4985 (0.1513–0.8457)
Swollen hock joint (*n* = 809)	0.5901 (0.4075–0.7727)
Swollen interdigital space (*n* = 975)	0.4422 (0.0366–0.8477)
SLH (*n* = 809)	0.5050 (0.4463–0.5638)
CPS (*n* = 987)	0.3996 (0.3534–0.4457)
DD	
Above heel (*n* = 979)	−0.0020 (−0.0041–−0.0000)
Around dew claws (*n* = 981)	0.8323 (0.6034–1.0000)
Skin above coronary band (*n* = 981)	−0.0014 (−0.0032–0.0005)
Interdigital space (*n* = 416)	0.6215 (0.5271–0.7159)
SDW (*n* = 495)	0.9651 (0.9257–1.0000)
HC (*n* = 495)	0.7990 (0.4135–1.0000)

SW = shifting weight; AWD = abnormal weight distribution; SHH = swollen heel, hock joint, or interdigital space; SLH = skin lesion on the lateral hock; CPS = claw position score; DD = digital dermatitis (acute M2 stage); SDW = short dorsal claw wall; HC = hyperextension of one claw; *n* = number of observations.

**Table 6:** The inter-rater reliability of the in-parlour scoring (IPS) indicators between an experienced and inexperienced observer is presented with a 95% confidence interval (in brackets) to demonstrate the reproducibility of this examination procedure. This analysis encompassed observations across eleven farms, collected during morning and evening milkings.

IPS Indicators	Inter-Rater Reliability
SW (*n* = 990)	0.3932 (0.2167–0.5696)
AWD (*n* = 1980)	0.4020 (0.3588–0.4452)
SHH	
Swollen heel (*n* = 3956)	0.4530 (0.1939–0.7122)
Swollen hock joint (*n* = 1661)	0.4061 (0.2557–0.5565)
Swollen interdigital space (*n* = 1805)	0.4513 (0.1918–0.7109)
SLH (*n* = 1661)	0.5343 (0.4928–0.5759)
CPS (*n* = 1977)	0.4253 (0.3950–0.4555)
DD	
Above heel (*n* = 1728)	−0.0037 (−0.0067–−0.0008)
Around dew claws (*n* = 1732)	0.3099 (0.1181–0.5017)
Skin above coronary band (*n* = 1731)	0.0000 (0.0000–0.0000)
Interdigital space (*n* = 1271)	0.3755 (0.2910–0.4600)
SDW (*n* = 990)	0.9463 (0.9115–0.9812)
HC (*n* = 990)	1.000

SW = shifting weight; AWD = abnormal weight distribution; SHH = swollen heel, hock joint, or interdigital space; SLH = skin lesion on the lateral hock; CPS = claw position score; DD = digital dermatitis (acute M2 stage); SDW = short dorsal claw wall; HC = hyperextension of one claw; *n* = number of observations.

**Table 7:** The distribution of locomotion scores (LCS), as defined by Sprecher et al. [8], for all cows included in the analysis is presented in the table below for each of the eleven farms. Locomotion scores 4 and 5 were combined to create a single category, designated as LCS ≥ 4. Percentages are provided in brackets.

Farm	LCS 1 (%)	LCS 2 (%)	LCS 3 (%)	LCS ≥ 4 (%)	Total
1	25 (65.8%)	13 (34.2%)	0	0	38
2	22 (62.9%)	13 (37.1%)	0	0	35
3	66 (60.6%)	40 (36.7%)	3 (2.8%)	0	109
4	38 (53.5%)	29 (40.8%)	4 (5.6%)	0	71
5	19 (55.9%)	13 (38.2%)	2 (5.9%)	0	34
6	4 (22.2%)	12 (66.7%)	2 (11.1%)	0	18
7	7 (28.0%)	12 (48.0%)	4 (16.0%)	2 (8.0%)	25
8	6 (18.8%)	22 (68.8%)	4 (12.5%)	0	32
9	6 (13.0%)	23 (50.0%)	11 (23.9%)	6 (13.0%)	46
10	3 (6.8%)	22 (50.0%)	14 (31.8%)	5 (11.4%)	44
11	1 (2.3%)	11 (25.6%)	21 (48.8%)	10 (23.3%)	43
**Total**	**197 (39.8%)**	**210 (42.4%)**	**65 (13.1%)**	**21 (4.2%)**	**495**

**Table 8:** Number and distribution of observations of the lameness indicators assessed by the experienced observer during the evening milking per farm and overall. The percentage is given in brackets.

In-Parlour Scoring Results—Evening Milking								
Farm	SW	AWD	SHH	SLH	CPS ^1^ 1 | 2 | 3	DD	SDW	HC
1	0	14	1	12	35 | 22 | 19	0	0	0
2	1	12	0	23	47 | 22 | 1	4	0	0
3	0	48	5	59	21 | 72 | 125	3	0	0
4	4	40	0	48	52 | 61 | 29	16	0	0
5	1	20	0	29	19 | 39 | 10	7	0	0
6	0	10	1	17	26 | 10 | 0	0 *	0	0
7	0	13	2	14	25 | 22 | 3	9	1	0
8	4	26	1	4	16 | 30 | 18	21	1	0
9	1	29	8	27	27 | 43 | 22	7	1	2
10	1	28	4	13	38 | 34 | 16	8	44	0
11	0	29	4	9	6 | 23 | 56 ^2^	14	0	0
**Total** **(% of total)**	**12** **(2.4)**	**269** **(54.3)**	**26** **(5.3)**	**255** **(51.5)**	**312 | 378 | 299** **(31.5 | 38.2 | 30.2)**	**89** **(18.0)**	**47** **(9.5)**	**2** **(0.4)**

SW = shifting weight; AWD = abnormal weight distribution; SHH = swollen heel, hock joint, or interdigital space; SLH = skin lesion on the lateral hock; CPS = claw position score; DD = digital dermatitis (acute M2 stage); SDW = short dorsal claw wall; HC = hyperextension of one claw. * Not endemic on this farm. ^1^ Two scores per cow. ^2^ One value is missing.

**Table 9:** Number and distribution of observations of the lameness indicators assessed by the experienced observer during the morning milking per farm and overall. The percentage is given in brackets.

In-Parlour Scoring Results—Morning Milking								
Farm	SW	AWD	SHH	SLH	CPS ^1^ 1 | 2 | 3	DD	SDW	HC
1	0	14	1	14	32| 29 | 15	0	0	0
2	1	16	0	20	55 | 15 | 0	4	0	0
3	0	51	7	37	41 | 94 | 83	10	0	0
4	2	36	0	35	60 | 62 | 20	4	0	0
5	1	14	0	30	17 | 42 | 9	4	0	0
6	2	10	1	15	26 | 8 | 2	0 *	0	0
7	1	13	2	12	9 | 30 | 11	11	1	0
8	4	25	1	2	26 | 25 | 13	23	3	0
9	0	25	3	17	19 | 49 | 24	10	0	3
10	0	28	5	10	43 | 30 | 15	10	44	0
11	2	32	3	17	9 | 26 | 51	15	0	0
**Total** **(% of total)**	**13** **(2.6)**	**264** **(53.3)**	**23** **(4.6)**	**209** **(42.2)**	**337 | 410 | 243** **(34.0 | 41.4 | 24.5)**	**91** **(18.4)**	**48** **(9.7)**	**3** **(0.6)**

SW = shifting weight; AWD = abnormal weight distribution; SHH = swollen heel, hock joint, or interdigital space; SLH = skin lesion on the lateral hock; CPS = claw position score; DD = digital dermatitis (acute M2 stage); SDW = short dorsal claw wall; HC = hyperextension of the claw. * Not endemic on this farm. ^1^ Two scores per cow. ^2^ One value is missing.

**Table 10:** The sensitivity, specificity, precision, and accuracy for the classifiers with the highest accuracy. Data are shown for the evening and morning milkings together and separately, as well as on a combined dataset comprising the maximum value of both.

	Sensitivity	Specificity	Precision	Accuracy	F1
Evening milking	0.24	0.96	0.56	0.84	0.33
Morning milking	0.19	0.99	0.80	0.85	0.31
Evening and morning milkings	0.19	0.98	0.67	0.84	0.29
Maximum value of evening and morning milking	0.24	0.96	0.56	0.84	0.33

**Table 11:** Number of ‘alarm lesions’ on the eleven farms at claw level documented during functional hoof trimming.

Farm	Ulcers (Sole, Toe, or Bulb Ulcers)	Toe Necrosis	White Line Abscess	Inflammatory Swelling of Coronet and Bulbs of Heel Associated with Deep Digital Sepsis	Interdigital Phlegmon (Foot Rot)	Acute (M2) Stage of Digital Dermatitis	DD- Associated Claw Horn Lesion
1	0	0	1	0	0	1	0
2	1	0	0	0	0	10	0
3	0	0	0	0	1	21	0
4	0	0	3	0	0	2	9
5	2	0	0	0	0	3	1
6	1	0	3	0	0	0	0
7	0	0	0	1	0	6	5
8	0	0	0	0	1	10	1
9	1	0	1	1	2	10	10
10	0	0	0	0	1	9	8
11	0	0	1	4	0	35	7
**Total**	**5**	**0**	**9**	**6**	**5**	**107**	**41**

## Data Availability

Further data are available at request from the corresponding author (see the Supplementary Material).

## Supplementary Materials

The following supporting information can be downloaded at: https://www.mdpi.com/article/10.3390/ani14192870/s1, Table S1. Mean sensitivity, specificity, precision, accuracy and F1 score and their standard deviations in parentheses for the four splits and the evening and morning milkings, together and separately, as well as on a combined dataset comprising the maximum value of both.
