# Supplementary material for: Evaluation of ‘In-Parlour Scoring’ (IPS) to Detect Lameness in Dairy Cows during Milking
Source: Animals (Basel). 2024 Oct 5;14(19):2870. doi: 10.3390/ani14192870 (PMC11482633; doi:10.3390/ani14192870)
Supplement: Supplementary file 1 [file animals-14-02870-s001.zip › animals-3190426-supplementary.pdf]

Table S1. Mean sensitivity, specificity, precision, accuracy and F1 score and their standard deviations in parentheses for the four splits and the evening and morning milkings, together and separately, as well as on a combined dataset comprising the maximum value of both.

	**Sensitivity**	**Specificity**	**Precision**	**Accuracy**	**F1 score**
Evening milking	0.15 (0.10)	0.96 (0.03)	0.58 (0.33)	0.82 (0.03)	0.22 (0.13)
Morning milking	0.12 (0.09)	0.97 (0.02)	0.39 (0.33)	0.82 (0.02)	0.18 (0.14)
Evening and morning milkings	0.16 (0.02)	0.97 (0.01)	0.53 (0.12)	0.83 (0.01)	0.24 (0.04)
Maximum value of evening and morning milking	0.12 (0.08)	0.98 (0.02)	0.61 (0.27)	0.83 (0.01)	0.18 (0.11)
